# Empyema Necessitans due to Interruption of Antituberculosis Treatment

**DOI:** 10.1155/2019/4810354

**Published:** 2019-05-29

**Authors:** H. N. Benaragama, J. Pushpakumara, Kamani P. Wanigasuriya

**Affiliations:** ^1^Colombo South Teaching Hospital-Kalubowila, Dehiwala-Mount Lavinia, Sri Lanka; ^2^Department of Medicine, Faculty of Medical Sciences, University of Sri Jayewardenepura, Nugegoda, Sri Lanka

## Abstract

Empyema necessitans is a rare presentation of tuberculous infection, commonly encountered in immunocompromised patients. The diagnosis can be challenging due to the paucibacillary nature of the condition and nonspecific symptoms. Identifying the organism in culture is the gold standard method of diagnosis. We describe a patient with chronic kidney disease, who developed empyema necessitans due to interruption of antituberculous therapy. Initially, he was started on antituberculous therapy based on a clinical diagnosis of smear-negative pulmonary tuberculous infection; this resulted in Stevens–Johnson syndrome needing a long course of steroid therapy. He later presented with a painful chest lump and was diagnosed as empyema necessitans. Finding the etiology for this rare presentation lead to a diagnostic dilemma, finally confirming the TB infection from the culture. This case highlights the importance of being aware of unusual complications of tuberculous infection in immunocompromised settings.

## 1. Introduction

Empyema necessitans (EN) is a rare manifestation of tuberculous (TB) infection. The exudative fluid in pleural cavity tracks down making fistula and manifests as a subcutaneous collection, where the patient can present with a chest wall lump [1, 2]. Confirming the diagnosis of a tuberculous infection is difficult because of the paucibacillary nature of the lesion [3, 4]. Treatment interruption to anti-TB treatment (ATT) can lead to this complication especially in immunocompromised patients [3, 5]. We encountered a patient who was treated with ATT for possible pulmonary TB infection, but treatments were interrupted during the initial round due to the development of Stevens–Johnson syndrome (SJS) for ATT, where he was treated with steroids withholding ATT. Later, he was readmitted with an anterior chest wall lump which was diagnosed as empyema necessitans. This case discusses the diagnostic dilemmas that were presented, due to the rare unusual presentation of TB empyema and the journey towards the final diagnosis. Combination of surgical drainage with ATT along with physiotherapy for improving pulmonary functions is the most effective method of treatment [6]. Almost all ATT can cause Stevens–Johnson syndrome (SJS) as an adverse effect [7, 8]. If it occurs, introducing ATT in a staged fashion with combination of first- and second-line drugs is recommended [8–10].

## 2. Case Presentation

A 34-year-old man presented with high-grade fever with chills and rigors and left-sided pleuritic type chest pain associated with a lump in the anterior chest wall. He was a patient with stage 5 chronic kidney disease and was on regular hemodialysis through an arteriovenous fistula during the past one year. He was awaiting kidney transplantation.

On examination, he was febrile with a temperature of 38.7°C. There were multiple enlarged tender axillary lymph nodes on the left side. The lymph nodes were around 0.5 cm and mobile and deep seated. He was dyspneic, breath sounds were reduced in the left lower zone of the lung with few crepitations. There was a tender lump (5 × 5 cm) on the left anterior chest wall. Other system examinations were found to be normal.

Approximately four months prior to the aforedescribed admission, he was investigated previously for fever, poor appetite, and loss of weight lasting for one-month duration. Clinical examination had revealed crepitation in the left lower lung base. Investigations done during that previous admission had revealed normal full blood count, erythrocyte sedimentation rate (ESR) of 93 mm in 1^st^ hour, and C-reactive protein at 23 mg/dl. Chest X-ray had shown evidence of left lower upper and lower lobe patchy opacities. He had been treated with IV co-amoxiclav 1.2 g three times daily empirically. As a further evaluation, contrast-enhanced computed tomography (CECT) of the chest was performed, which had revealed poorly defined small nodules and tree-in-bud opacities in the left lower lobe with focal consolidation in the apical segment of the left upper lobe in keeping with chronic lung infection. A small well-defined enhancing subpleural nodule with speculated margin in the posterior segment of the left lobe and few hilar and pretracheal enlarged lymph nodes were also seen. The Mantoux test had been 20 mm. Sputum direct smear for acid-fast bacilli (AFB) and culture had been negative. A tentative diagnosis of tuberculous infection had been done, and he was started on antituberculous therapy (ATT), namely, rifampicin, ethambutol, pyrazinamide, and isoniazid. After two weeks of ATT, the patient had developed Stevens–Johnson syndrome (SJS), and ATT was discontinued. His symptoms had improved with prednisolone 60 mg daily with tapering over the next preceding month, but ATT was not recommenced due to uncertainty of the diagnosis, because by that time, patient's symptoms had been resolved, and the sputum for tuberculosis (TB) culture was reported as negative.

**Figure 1:**
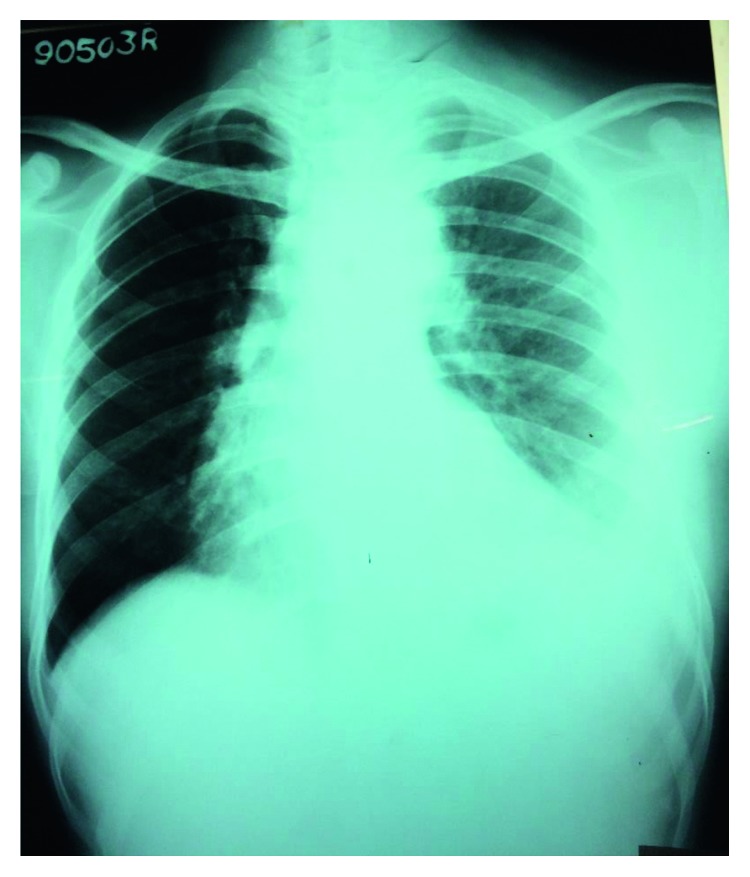
Chest X-ray showing left-sided consolidation and an effusion.

Investigations done during the latter admission revealed white cell count of 10,000 with neutrophils of 89%, lymphocytes of 10%, eosinophils of 0.2%, hemoglobin at 7.2 g/dl, mean corpuscular volume of 93 fl, and platelets 278 × 10^3^. The erythrocyte sedimentation rate (ESR) was 101 mm in the first hour, and C-reactive protein was 311 mg/dl. Chest radiograph showed left lower zone effusion with consolidation (see Figure 1). Considering the history and the high inflammatory markers with X-ray changes, a possible diagnosis of left lower lobe pneumonia with parapneumonic effusion was considered. Initially, he was treated with IV co-amoxiclav 1.2 g three times daily. Sputum for gram stain, acid-fast bacilli, was found to be negative. Sputum for pyogenic culture also did not isolate any organism after 72 hours of incubation. Ultrasound-guided aspiration of the pleural fluid was carried out and aspirated purulent blood-stained fluid. Lymph node fine needle aspiration or biopsy was not considered for several reasons. First, as it was situated on the left axilla, where the patient had his fistula made for the hemodialysis. Second is that the lymph nodes were found to be too small for sampling. Third was since they were deep seated, and it was difficult to gain access, and lastly, because of the possibility of arriving at a diagnosis by evaluating the effusion where access is easily gained.

**Figure 2:**
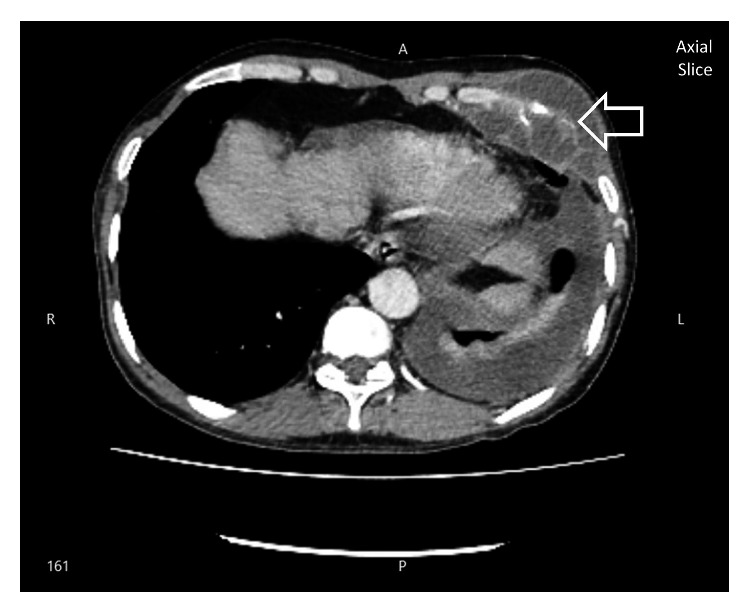
CECT chest showing left side empyema necessitans with rib destruction (arrow).

**Figure 3:**
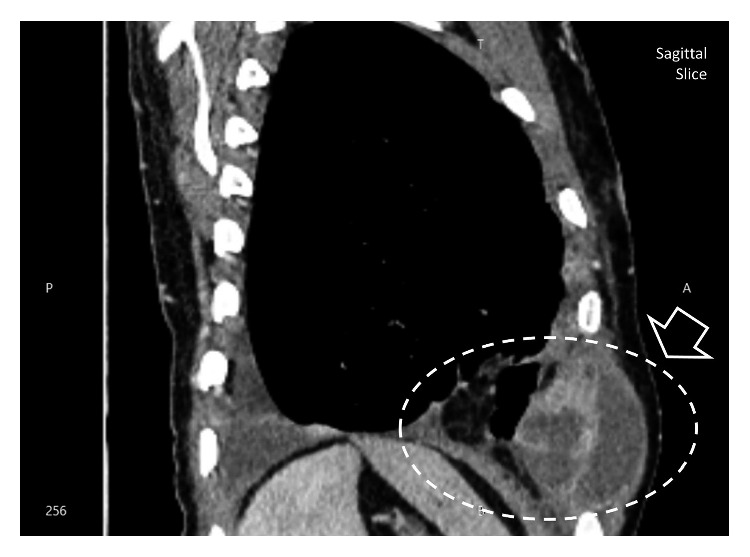
CECT chest exudative fluid in pleural cavity tracks down making fistula.

The aspirate was analyzed with gram stain and AFB stain, which were negative. Pyogenic culture of the aspirate remained sterile after 72 hours. Full report of the aspirated fluid revealed 90% polymorphs and 10% lymphocytes. Lactate dehydrogenase (LDH) was 12,738 IU/l. Adenosine deaminase level (ADA) was 431 *μ*/l. Because of the high inflammatory markers and the neutrophil predominance, he was treated with intravenous (IV) meropenem 500 mg twice daily and with IV clindamycin 500 mg twice daily for two weeks. But since the ADA was very high, with relation to the past history, underlying tuberculosis infection was also considered. Therefore, aspirate fluid was sent for TB culture. While awaiting TB culture, subsequent CECT revealed empyema necessitans with destruction of the left side anterior upper rib associated with left pleural and mediastinal lymphadenopathy with the most probable infection of tuberculosis (see Figures 2 and 3).

The patient was referred to the thoracic surgical team, and they carried out incision and drainage of the lump since the effusion was minimal by that time with the drainage and the treatments. Abscess wall histology did not reveal caseating granulomas. The abscess fluid culture isolated 19 colonies of *Mycobacterium tuberculosis* organism after one month of incubation; hence, tuberculous empyema necessitans was confirmed.

## 3. Discussion

Tuberculous (TB) infection is a globally common infection with a significant morbidity and mortality, considered as the ninth leading cause of death among HIV-infected and immune-compromised patients globally, and has found to account for >90% deaths of such patients in developing countries [5, 11]. The disease may only manifest in immunocompromised states such as HIV infection, diabetes mellitus, and malnutrition, because of the dormant behavior of tuberculous bacteria [3, 5]. Patients with chronic kidney disease who are on regular hemodialysis are also at a higher risk of infection than the normal population (6.9- to 62.5-fold higher) due to deficiency in cellular and humoral immunity [3]. It is considered about 33% of the population will have latent TB [1], and 15% of patients with TB infections will present as extrapulmonary infections [12]. EN is a rare manifestation of extrapulmonary TB (EPTB). The exudative fluid in the pleural cavity tracks down making a fistula and manifests as subcutaneous collection, where the patient can present with a chest wall lump. Although it is commonly seen with tuberculous infection, pyogenic bacteria, actinomycosis, blastomycosis, and some malignancies are other etiological agents of empyema necessitans [1–3].

Anterior chest wall between midclavicular and anterior axillary line and between the second and sixth intercostal spaces is the commonest site to develop EN [8]. Other recognized sites are bronchus, vertebral column, diaphragm, breast, mediastinum, retroperitoneum, esophagus, pericardium, flank, or groin [8].

Due to the paucibacillary nature of the tuberculous bacteria, and nonspecific symptoms and signs, confirming the diagnosis of tuberculosis is difficult, especially in EPTB [3, 4]. Direct isolation of tuberculous bacteria requires collecting the appropriate sample, mostly with invasive procedures [5]. Even after the appropriate sample collection, the sensitivity of most tests is very low. Out of all the investigations, isolation of bacterium through TB culture is considered the gold standard, but it is time-consuming [5]. Sputum for acid-fast bacilli was repeatedly negative in our patient, and the sputum culture was also negative repeatedly for mycobacteria, leading to a delayed diagnosis.

During the second admission of the patient, although the CECT, high levels of LDH, and ADA in exudates were suggestive of TB infection, we did not initiate ATT due to several factors. Firstly, the pleural fluid aspirate full report revealed evidence towards a bacterial infection because of the exudative effusion and neutrophil predominance. High inflammatory markers in blood also supported this. Secondarily, due to the history of serious drug reaction to ATT, and lastly, that the fever settled with antibiotic treatments, even though the lump persisted. This led to a diagnostic dilemma, whether it is TB with empyema or due to other bacterial pathogens leading to parapneumonic effusion. This dilemma caused delay in starting ATT. Finally, the chest wall abscess culture isolated 19 colonies of *Mycobacterium tuberculosis* organism, giving the confirmation of diagnosis of tuberculous infection after about 8 months from the initial presentation.

Reported cases in the literature describe patients presenting with chest wall lump of EN in the initial presentation in immunocompromised patients or with a more virulent infection in immunocompetent patients [1–3, 12–17]. EN due to tuberculous infection is mostly reported among immunocompromised patients. Some reported cases describe it as a late manifestation of EPTB infection in patients who had history of pulmonary or EPTB, even though treatments had completed many years ago [2, 14]. The articles describe this was probably because of paucity of TB organism as well as clinical symptoms in geriatric populations [2]. EN has been reported among immunocompetent patients also with the most common cause being identified as the TB bacterium [1, 13, 16, 17]. Other reported cases among immunocompetent patients were because of the virulence of organism such as *Staphylococcus aureus* (MSSA and MRSA) and Actinomycosis [1, 2]. There is one reported case of EN due to TB infection in a patient with peritoneal dialysis, because of the lack of cellular and humoral immunity [12]. Even though the Mantoux test was strongly positive in the initial presentation, which is a good indicator of the host's immunity, treatment with prednisolone supported with regular hemodialysis for chronic kidney disease must have accelerated the immunocompromised state in our patient. Previous interruption of ATT with compromised immunity, had led to this rare presentation of TB infection in our patient.

TB effusion is characterized by lymphocytic predominance, high LDH, and ADA levels [17]. For the formation of granuloma, the acute inflammatory response should be followed by immune-mediated, lymphocytic-driven chronic inflammation [17]. Neutrophil predominance is also seen among patients with uncommon presentation which was often mistakenly diagnosed as parapneumonic effusion as in our patient [9, 17]. A study carried out in South Korea reports that approximately 10% of diagnosed patients with pleural TB had neutrophil predominance with high inflammatory markers and high LDH [17]. The conclusion for such presentation was explained as presence of high inflammation in the early stage of the disease driven by neutrophils. The study also reveals that neutrophil predominance patients' infectivity is more, because higher yield of positive cultures of TB had been observed when compared to the lymphocytic predominance effusion [17]. Presence of other concomitant bacterial infections is also another possibility for the neutrophil predominance effusion [9].

Our patient's pleural effusion was also a neutrophil predominant effusion with high LDH and ADA level. His fever and inflammatory markers responded to IV antibiotics. He had been treated with steroids and was tailed off. The possible explanation is that he must have been in the early inflammatory phase of the TB infection, which gave rise to neutrophil predominant effusion and absent granuloma formation in the abscess wall, with concomitant bacterial infection. Gram stain and cultures did not isolate any other bacterial pathogens probably because of prior antibiotic use before obtaining the cultures.

Initiating treatment was challenging because of the SJS for ATT and as the diagnosis of TB infection was in debate initially. Therefore, in our patient, we waited until the confirmation of TB infection was made through culture to start ATT. There are case reports and is a cohort study carried out by Chulalongkorn University of Bangkok, Thailand, where it describes that ATT should be introduced individually with gradual desensitization to recognize the responsible drug for SJS [8–10]. This method was followed with this patient, first starting the second-line anti-TB drugs with gradual desensitization and introducing typical ATT drugs with least possible drug to develop SJS. He tolerated ofloxacin, ethambutol, and pyrazinamide but developed reaction to INAH. Therefore, the three former drugs were continued as ATT. Steroid use in tuberculous infection is recommended to prevent constrictive pericarditis, hydrocephalus, focal neurological deficits, pleural adhesions, and intestinal strictures especially in immunocompromised HIV patients but with combination of ATT [18]. Treatment of EN requires surgical drainage, mostly thoracoplasty and ATT [19]. Most reported cases were managed with thoracoplasty, and there is one reported case where it was managed medically by starting early ATT [16]. In our patient also, we managed to do a simple incision and carryout draining of pus. He improved with ATT and lung expanding physiotherapy and did not develop further abscess formation or pleural adhesions.

This serious and rare complication of the TB infection would have prevented if the ATT was reintroduced in a gradual desensitization method after resolving of SJS.

## 4. Conclusion

Empyema necessitans (EN) is a rare complication of tuberculous infection. Patients with chronic kidney diseases who are on regular dialysis are more vulnerable for this infection. Treatment interruption for TB infection will lead to rare but serious complications. Neutrophil predominance in a TB effusion is an atypical presentation and should be considered if there is high suspicion of TB clinically. Surgical drainage combined with ATT is the most effective method of treatment for EN. SJS to ATT is a well-known complication, and introduction of individual second-line drugs with gradual desensitization is recommended.
